# Guided assembly of nanoparticles on electrostatically charged nanocrystalline diamond thin films

**DOI:** 10.1186/1556-276X-6-144

**Published:** 2011-02-14

**Authors:** Elisseos Verveniotis, Alexander Kromka, Martin Ledinský, Jan Čermák, Bohuslav Rezek

**Affiliations:** 1 Institute of Physics ASCR, Cukrovarnicka 10, 16253, Prague 6, Czech Republic

## Abstract

We apply atomic force microscope for local electrostatic charging of oxygen-terminated nanocrystalline diamond (NCD) thin films deposited on silicon, to induce electrostatically driven self-assembly of colloidal alumina nanoparticles into micro-patterns. Considering possible capacitive, sp^2 ^phase and spatial uniformity factors to charging, we employ films with sub-100 nm thickness and about 60% relative sp^2 ^phase content, probe the spatial material uniformity by Raman and electron microscopy, and repeat experiments at various positions. We demonstrate that electrostatic potential contrast on the NCD films varies between 0.1 and 1.2 V and that the contrast of more than ±1 V (as detected by Kelvin force microscopy) is able to induce self-assembly of the nanoparticles via coulombic and polarization forces. This opens prospects for applications of diamond and its unique set of properties in self-assembly of nano-devices and nano-systems.

## Introduction

Electrostatic charging of surfaces is widely used in a variety of technological processes. It improves wetting of plastics for painting, it is employed in electronics, e.g., in detectors or memory devices, and it is used in printers and copiers for toner positioning on paper. In this context electrostatic charging has been also explored as an effective method for guiding self-assembly of micro- and nanosized elements on insulating materials [1-3]. Electrostatic charging can be generated by various methods (laser, ion, or electron beam illumination, diverse electrodes, etc.). Charged patterns of sub-micrometer dimensions can be created using nanometer-sized probes, such as those employed in atomic force microscopy (AFM) [4,5].

A large variety of materials have been applied for electrostatic charge storage: semiconductors [4] including amorphous silicon [5] as well as dielectric materials such as polytetrafluoroethylene and poly(methyl methacrylate) [6]. Detection and understanding of electrostatic charging of diamond is crucial for many diamond-based electronic applications from detectors to field-effect transistors, batteries, silicon on diamond systems as well as for electrostatically guided assembly. This is because diamond as a semiconductor material can, for instance, be used for device fabrication [7], for passive and active bio-interfaces [8,9], and can be deposited on diverse substrates in nanocrystalline form [10]. From the electronic point of view, diamond is a wide band gap semiconductor (5.5 eV). Nevertheless, it can be transformed into p- or n-type semiconductor by boron [11] or phosphorus [12] doping, respectively. Intrinsic diamond is generally electrically insulating and transparent for visible light. Only when the intrinsic diamond is hydrogen-terminated (H-diamond), a thin (<10 nm) conductive layer is formed close to the diamond surface (surface conductivity) under ambient conditions [13]. While this feature attracted considerable interest and research effort in the past [14], research on electronic properties of highly resistive oxygen-terminated intrinsic diamond (O-diamond) has been limited. It was related mostly to applications in radiation detectors [15], UV detectors [16], or field-effect transistors [17,18].

As regards local and intentional electrostatic charging, diamond has been only little investigated [19-21] even though it exhibits a unique set of properties for applications as described above. Both positive and negative persistent potential changes were observed on nanocrystalline diamond (NCD) [19], unlike in silicon thin films [5]. This has been attributed to the capacitor-like behavior of the NCD films [19]. Comparing charging of NCD films prepared on gold [19] and silicon [20] substrates demonstrates that the charging is not due to the substrate itself as could be argued in the case of silicon substrates. The charging has been also shown to be more effective when the NCD films contain more sp^2 ^phase [21]. Surprisingly, the charging is spatially homogeneous and not confined to grain boundaries where most of the sp^2 ^is localized [20]. Yet maximal induced electrostatic potential contrast has been reported to be varying by up to 400 mV depending on a position on the sample [20]. This may depend on the local material properties as well as actual tip condition.

In this article, we apply local electrostatic charging of oxygen-terminated NCD films to induce electrostatically driven self-assembly of colloidal nanoparticles into micro-patterns. Considering possible capacitive, sp^2 ^phase, and spatially related contributions to charging, we employ films with sub-100 nm thickness, and about 60% relative sp^2 ^content, probe their material uniformity, and repeat experiments at various positions across the films to induce as much potential contrast as needed for the self-assembly.

## Materials and methods

NCD films were prepared by microwave plasma chemical vapor deposition using the following parameters: substrate temperature 820°C, deposition time 16 min, microwave plasma power 900 W, CH_4_:H_2 _dilution 3:300. Resulting thickness was 74 nm as measured by ellipsometry. The substrates were 5 × 10 mm^2 ^conductive p-doped silicon wafers nucleated by water-dispersed detonation diamond powder of 5 nm nominal particle size (NanoAmando, New Metals and Chemicals Corp. Ltd., Kyobashi) using an ultrasonic treatment for 40 min. After the deposition, the diamond films were oxidized in r.f. oxygen plasma (300 W, 3 min) [22].

Localized charging was performed by scanning in contact mode with an atomic force microscope (N-TEGRA system by NT-MDT). Conductive, diamond-coated silicon probes were used (DCP11 by NT-MDT). Applied contact forces were ~100 nN. The bias voltage was applied to the tip while the silicon substrates were grounded. An external voltage amplifier (HP 6826A) was connected to the cantilever and controlled by the AFM software via a signal access module, to apply voltages within the range of ±25 V (the potential contrast is saturated at these voltages [20]). The scan speed was always 10 μm/s. Kelvin force microscopy (KFM) was then used to detect potential differences across the sample [23]. The KFM potential values and differences are given here as measured, not with respect to the vacuum level. Relative humidity and temperature during all AFM experiments were in the ranges of 20-32% and 22-26°C.

For resolving typical grain size, shape, and film homogeneity, scanning electron microscopy (SEM) was applied (eLine by Raith, secondary electron detector, accelerating voltage 10 kV, working distance 8 mm). Micro-Raman spectroscopy (inVia by Renishaw, HeCd laser, λ = 325 nm, objective 40×, spot diameter 2 μm) was employed to determine the material properties and uniformity across the films.

For achieving directed self-assembly of nanoparticles, a charged sample was immersed vertically into a colloidal emulsion for 10 s. The sample was then let to dry in air for 5 min. The emulsion was prepared by putting 300-500 μl of the aqueous suspension containing the nanoparticles (alumina of 50 nm nominal size, particle concentration 15%, Buehler, USA) into 5 ml of an insulating fluorocarbon solution (Fluorinert FC-77, 3M Company, USA) and ultra-sonicating the mixture for 20 s. Since the two liquids do not mix, ultrasonication provided the means for creating emulsion with microscopic colloidal droplets [3]. FC-77 was selected due to its inertness, letting the charged features to maintain their electrical potential even after immersion, and allowing electrostatic forces to reach relatively far into the emulsion (~1 μm).

## Results

**Figure 1:**
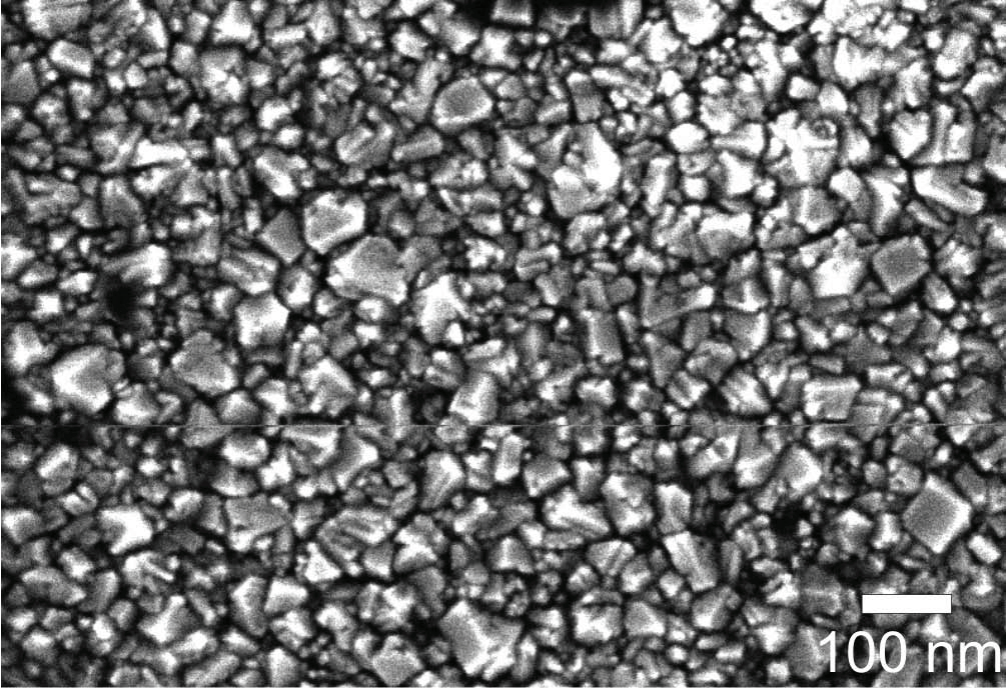
**Micrograph from scanning electron microscopy on the employed nanocrystalline diamond thin films**.

Figure 1 shows a typical SEM image of an NCD sample. The NCD film appears continuous and uniform in surface morphology. There are smaller and bigger grains with resolvable crystalline facets. Average size of the grains is 53 ± 35 nm as evaluated from the SEM images. SEM investigation across the whole sample showed very similar structure, which indicates that our film is spatially uniform. The root-mean-square (RMS) roughness measured by AFM is about 5 nm.

**Figure 2:**
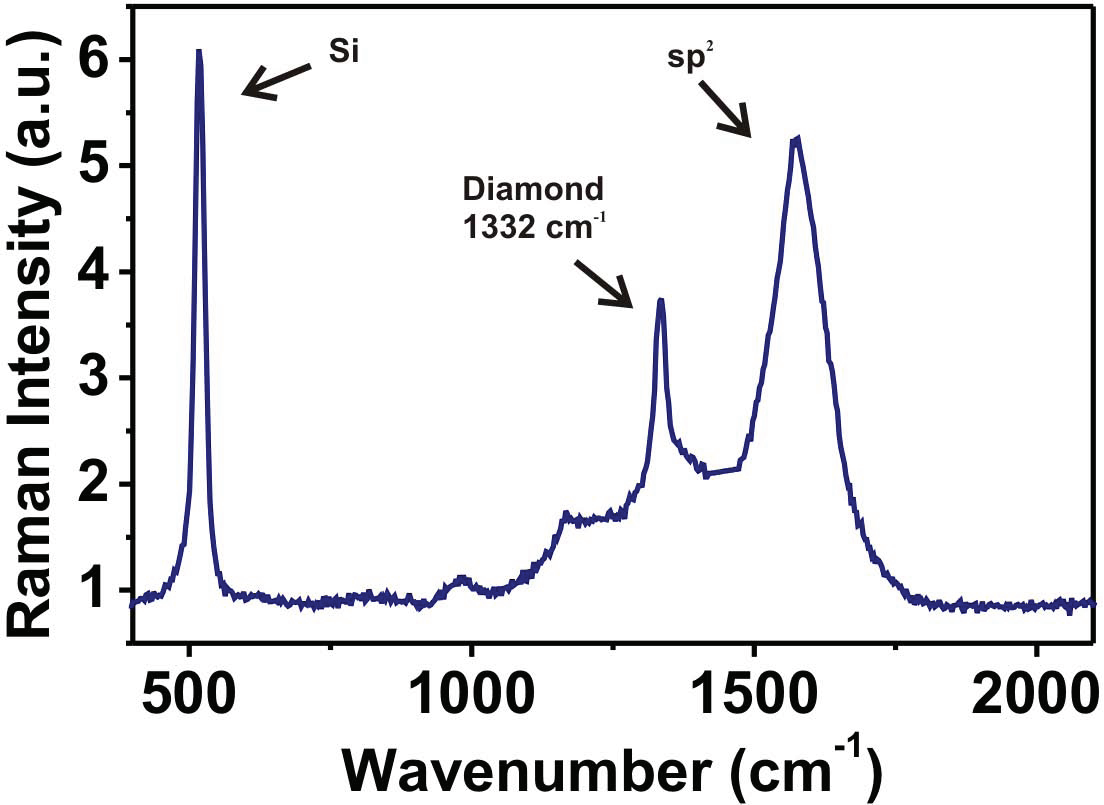
**Typical micro-Raman spectrum (UV laser, λ = 325 nm) on the employed nanocrystalline diamond thin films**.

Figure 2 shows a typical micro-Raman spectrum of the NCD film. It exhibits clear sp^3 ^peak at 1332 cm^-1 ^indicating diamond character. Note that repeating the measurement on different spots across the sample indicated slight differences in the sp^2 ^(graphitic) phase content. The calculated relative percentage of the sp^2 ^phase from Raman spectra [[*I*_sp2_/(*I*_D _+ *I*_sp2_)] * 100] [24] is ranging between 58 and 60%.

**Figure 3:**
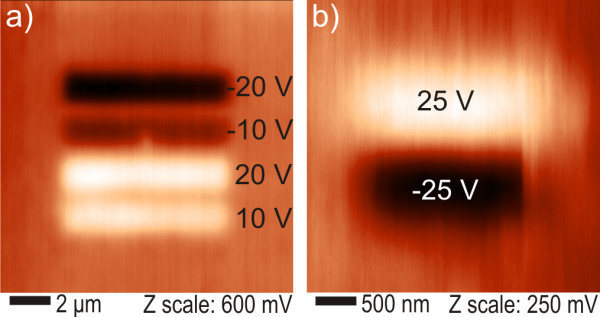
**Kelvin force microscopy surface potential maps after typical charging experiments**. **(a) **up to ±20 V and **(b) **at ±25 V. Charging voltages are indicated near each stripe pattern.

Figure 3 shows KFM surface potential maps after the typical charging experiments. In Figure 3a we applied the charging voltage of 10, 20, -10, -20 V in an 8 μm^2 ^area during contact mode AFM scan, while scanning horizontally and with slow scan direction from the bottom to the top. The maximum potential values with respect to the background for the charging voltages of ±20 V are 210 mV for the positive and -390 mV for the negative polarity. Figure 3b shows the KFM map after charging with ±25 V in another 2 μm^2 ^area. Those voltages are at or above the saturation threshold of charging [20]. Yet the maximum potential values are only 110 mV for the positive and -140 mV for the negative polarity.

**Figure 4:**
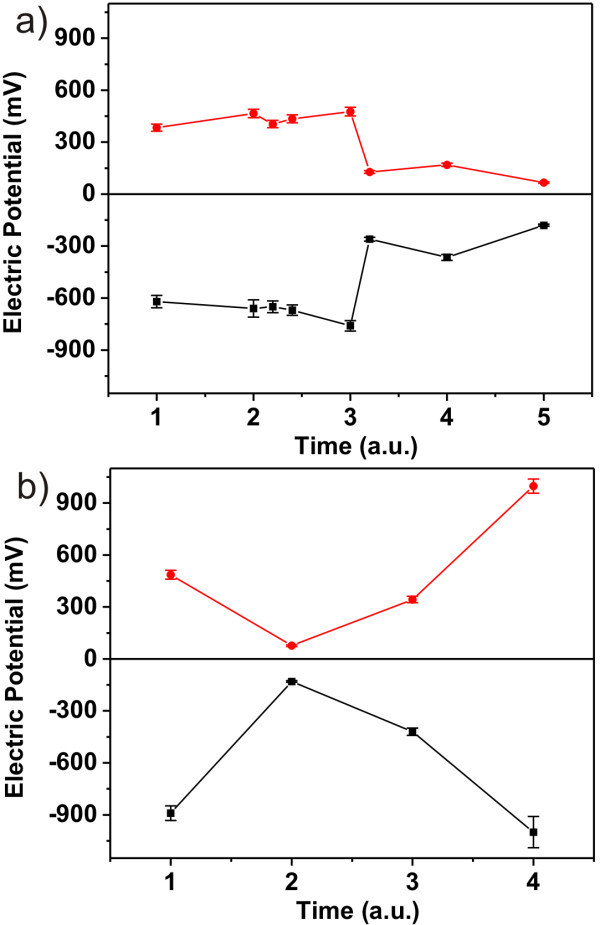
**Surface potential shifts after electrostatic charging for positive and negative polarity**. The data points correspond to average potential within the individual stripes that were charged using **(a) **±20 V or **(b) **±25 V. Positive and negative data points at the same *x*-value were obtained from a charging experiment and KFM in one scan frame. Only in the case of *x *= 4 in **(b) **the patterns were charged in separate frames. The *x*-axis values between two integer values in **(a) **correspond to experiments conducted within the same day.

**Figure 5:**
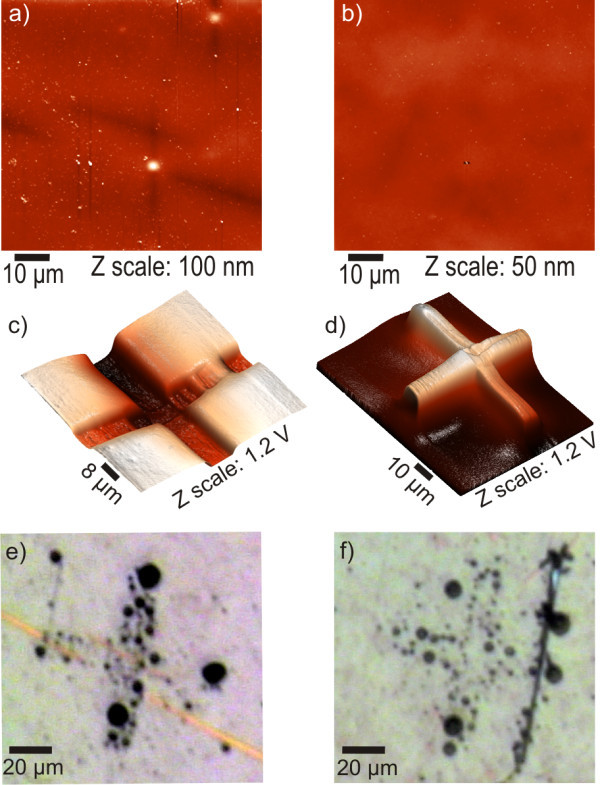
**Local topography of typical work areas on the NCD thin films**. **(a, b) **AFM morphology on charged areas. **(c, d) **Corresponding KFM of electrostatically charged crosses on the nanocrystalline diamond thin film using **(c) **negative and **(d) **positive voltage. **(e, f) **Optical microscope pictures of the charged crosses after immersion to emulsion containing alumina nanoparticles.

The maximum achievable potential shift in each polarity was varying when the experiment was repeated (inherently at another position on the sample). This is illustrated in Figure 4a,b, where we can see the total potential contrast varying from 230 to 2000 mV. The data points in Figure 4 correspond to average potential within the individual stripes that were charged using ±20 V (Figure 4a) or ±25 V (Figure 4b). The *x*-axis values between two integer values in Figure 4a correspond to experiments conducted within the same day. Positive and negative data points at the same *x*-value were obtained from a charging experiment and KFM in one scan frame such as the ones in Figure 3. Only in the case of *x *= 4 in Figure 4b the patterns were charged in separate frames (shown in Figure 5c,d). On the graphs we can also observe that charging with ±25 V does not always result in higher potential. Even though we did obtain the highest contrast to date with this voltage (*x *= 4, Figure 4b), there are features charged with ±20 V that exhibit higher potential than others charged with ±25 V (e.g., *x *= 2-3 in Figure 4a vs. *x *= 2, 3 in Figure 4b).

By experimenting repeatedly we were able to achieve ≥1 V contrast in each polarity on our sample and we used those patterns for self-assembly. Figure 5a-d shows the AFM and KFM images of such patterns in each polarity charged with -25 and 25 V (corresponds to *x *= 4, Figure 4b). The dimensions of the cross arms are 10 × 80 μm. Maximum amplitude of the charged patterns is 1.2 V (average = 1 V) in each polarity. The centers of the crosses show slightly higher potential compared to the rest because they were charged twice (horizontally and vertically). The potential is not double, though, since the charging exhibits saturation as reported before [20]. AFM in such large scale confirms the homogeneity of our samples and excludes possible external contributions to the observed electric potential shifts (i.e., topographical variations). Note that structural details are not resolvable because the size difference between a typical grain and the full scan shown here is at least three orders of magnitude (18-88 nm vs. 80 μm).

In Figure 5e,f we can see optical microscope images of the charged crosses after immersion to the solution containing the alumina nanoparticles. The nanoparticles assembled preferentially on the crosses. Their arrangement is determined by the polarity of the particular charged cross. Negative potential produced a filling effect (Figure 5e), where the assembly occurred on the charged area. Positive potential leads to a decorative effect (Figure 5f), where the nanoparticles attached predominantly on the cross edges. Charged patterns having potential contrast below 1 V did not lead to preferential assembly of the nanoparticles.

## Discussion

In order to generate self-assembly of nanoparticles on charged areas, the electrostatic forces must be high enough to attract particles from the solution and promote assembly. In various charging instances identical to the one shown in Figure 5 but with less charged potential (up to 800 mV average potential) self-assembly was not possible. Therefore, we assume that potential differences below 1 V are insufficient to generate the self-assembly. The contrast of 1.2 V versus the uncharged background was already sufficient to generate self-assembled patterns even though it is still considerably lower than the potentials typically used in the case of dielectric materials (3-5 V) [1-3]. The assembled nanoparticle concentration is higher in the top component of the cross in Figure 5c as it exhibits slightly higher potential compared to the other two components on which particles did assemble. Furthermore, the lower charge in the right element of the same cross (600 mV) leads to missing particles in that region. Combination of positively and negatively charged regions [20] may improve definition of the self-assembled pattern, but it will not increase the electrostatic force itself needed for assembly. Hence the properties and charging process of NCD film have to be optimized to achieve contrast ≥1 V versus the surrounding surface of the film.

The different behavior per polarity can be explained from the fact that the nominally uncharged nanoparticles got positively charged (including their aqueous shell around them) when emulsified in the FC-77. This is due to the relative dielectric constant ε_r _difference between the materials (9.9 vs. 1.86), as materials with higher ε_r _tend to charge positively when brought in contact with other materials having lower ε_r _[3]. Hence, the positively charged nanoparticles cover negatively charged areas via coulomb interaction (see Figure 5e). The edge decoration observed in Figure 5f is due to the attachment of non-charged or weakly charged nanoparticles that are attracted via polarization effects to the places exhibiting the highest electrostatic field gradient.

It is noticeable that in the present case the selectivity of nanoparticles toward negative versus positive patterns is low. It indicates generally low charge on the nanoparticles in the emulsion. Optimizing the emulsion and/or nanoparticles may improve the selectivity toward specific charge polarity on diamond as reported for dielectric materials [3].

Another problem is the large variation in the potential contrast on the NCD films. There are various factors that can influence the charging and lead to the observed potential contrast variations in different experiments/positions under otherwise same experimental conditions. First factor is the ambient environment. Humidity can affect the size of the meniscus formed between the AFM tip and the sample while scanning under ambient conditions [25]. This can influence the area over which the voltage is applied, possibly altering electric field and current density, current path, as well as capacitance. Ambient temperature variations may also influence the electrical behavior of the system by moving the conduction threshold [26]. Second, the tip-sample junction properties have to be considered. Change in the electrical contact between the tip and the sample could be caused even during the same scan if the surface under investigation is rough [27]. In addition, the AFM tip can be abraded due to scanning. This could lead to local removal of the conductive-diamond coating of the tip, bringing the sample in contact with the residual SiO_2 _at the very tip end [28]. This may cause a drop in the applied voltage, which would result in lower voltage across the diamond itself. Third, the cross-sectional morphology of the diamond film may also play a role. As the relative sp^2 ^content of the charged film is believed to be the governing factor toward effective charging [21], local accumulation of very small grains under the surface on the specific area being charged may lead to an increase in the local sp^2 ^content (more grain boundaries). This could increase the potential contrast.

In our case, the range of relative humidity and temperature, under which the experiments were conducted, was within 12% and 4°C, respectively. The samples were also relatively flat (5 nm RMS) and uniform. Local material differences based on the micro-Raman spectra are within 2% of relative sp^2 ^content. We assume that this is not enough to explain variation by almost an order of magnitude in the potential contrast. Hence the tip condition may be the important factor. Even diamond-coated tips may not be durable enough under hard conditions [29]. In spite of the above-mentioned problems, we were able to demonstrate the feasibility of self-assembly on diamond. Understanding and systematically achieving high potential contrast on NCD is only a matter of future research.

## Conclusions

We have demonstrated successful electrostatically guided self-assembly of alumina nanoparticles into micro-patterns on NCD thin films. We have shown that the electrostatic potential contrast on the NCD films induced by charging must be ≥ ± 1 V to generate the self-assembly. In spite of variations in the maximum potential contrast (0.1-1.2 V) - most likely mainly due to a changing quality of tip-surface junction under otherwise same conditions - NCD films rich in sp^2 ^(about 60% relative content) employed in this study were able to retain the high enough potential contrast and consequently induce the self-assembly process. This opens prospects for applications of diamond and its unique set of properties in self-assembly of nano-devices and nano-systems.

## Abbreviations

AFM: atomic force microscopy; KFM: Kelvin force microscopy; NCD: nanocrystalline diamond; RMS: root-mean-square; SEM: scanning electron microscopy.

## Competing interests

The authors declare that they have no competing interests.

## Authors' contributions

EV carried out the AFM/KFM measurements, performed the charging/self-assembly and drafted the manuscript. AK performed the nucleation and deposition of the NCD thin films. ML performed the Raman measurements. JČ participated in the optimization of the AFM/KFM methodology. BR conceived the study, participated in its design and coordination and edited the manuscript.
